# How to evaluate the risk/benefit of trimodality therapy in locally advanced non-small-cell lung cancer

**DOI:** 10.1038/sj.bjc.6603751

**Published:** 2007-05-01

**Authors:** H Kunitoh, K Suzuki

**Affiliations:** 1 Department of Internal Medicine and Thoracic Oncology, National Cancer Center Hospital, 5-1-1 Tsukiji, Chuo-ku, Tokyo 104-0045, Japan; 2 Department of Thoracic Surgery, National Cancer Center Hospital, 5-1-1 Tsukiji, Chuo-ku, Tokyo 104-0045, Japan

**Keywords:** trimodality, preoperative chemoradiotherapy, stage III NSCLC, superior sulcus tumour

## Abstract

The trimodality approach represented by concurrent chemoradiotherapy followed by surgical resection is a highly effective, but potentially toxic therapy for locally advanced non-small-cell lung cancer (NSCLC). In this review, we discuss the current status of this therapy in patients with mediastinal node-positive (N2) stage III NSCLC or superior sulcus tumor, and present an overview of the principles for optimisation of the risk/benefit. Numerous clinical questions remain, and enrolment of patients into well-designed clinical trials should be encouraged.

Lung cancer is the leading cause of death from cancer in Japan as well as in other industrialised nations. Non-small-cell lung cancer (NSCLC) accounts for about 80% of all cases.

About one-third of all cases of NSCLC present with locally advanced, stage IIIA/IIIB disease, most frequently with mediastinal node involvement (N2). Surgical resection was employed as ‘standard’ therapy for relatively less advanced ‘resectable’ cases with N2 NSCLC; however, the prognosis was not favourable (Suzuki *et al*, 1999). For more advanced, ‘bulky’ unresectable N2 disease, neither satisfactory local control nor satisfactory suppression of micrometastases was achieved with definitive thoracic radiotherapy (Schaake-Koning *et al*, 1992; Dillman *et al*, 1996).

Systemic chemotherapy was introduced two decades ago for cases with stage III N2 NSCLC (Le Chevalier *et al*, 1991; Schaake-Koning *et al*, 1992; Sause *et al*, 1995; Dillman *et al*, 1996), aimed at both eradication of micrometastases and improvement of local control. A multimodality approach to treatment, for example, systemic chemotherapy combined with definitive local therapy, is now the most preferred approach in the battle against stage III NSCLC. In particular, trimodality therapy, combining surgery, radiotherapy and chemotherapy, has been intensively investigated often with promising results. The sequence of ‘trimodality’ therapy that has most often been employed is induction chemoradiotherapy, followed by surgical resection, with or without consolidation chemotherapy. Although such therapy has sometimes been shown to be highly effective, it is also highly toxic, with a reported treatment-related death rate of as high as 10%. Careful evaluation of the risk/benefit ratio of such therapy is thus indispensable.

On the other hand, a small subset of NSCLC called superior sulcus tumour (SST) or Pancoast's tumour, in which the tumor is located in the superior sulcus and involves structures at the thoracic inlet, has posed a challenging problem for surgeons, radiation oncologists and medical oncologists alike, ever since it was first discovered (Rusch *et al*, 2001). However, the trimodality approach mentioned above has been shown to be associated with a relatively more favourable risk/benefit ratio in this subset of patients, for whom it currently appears to be the treatment of first choice (Rusch *et al*, 2001; Kunitoh *et al*, 2003).

In this study, we review the current data on the use of the trimodality approach in the treatment of patients with locally advanced NSCLC, and also discuss means to optimise the treatment using this approach.

## RATIONALES FOR TRIMODALITY THERAPY

Although the strongest rationale for the use of the trimodality therapy stems from the promising results of clinical phase II data, upfront systemic chemotherapy offers several practical as well as theoretical advantages (Pisters *et al*, 2000). Early introduction of systemic therapy may be expected to lead to early control of micrometastases. Response to the therapy can be easily assessed by radiographic imaging, which can help physicians avoid unnecessary, ineffective therapy. Visualisation of the response could motivate the patients to accept additional potentially toxic therapy, which cannot be said for postoperative chemotherapy. In addition, as compared to the postoperative status, pre-operative patients are usually in a much fitter state for chemotherapy.

Addition of radiotherapy to preoperative chemotherapy should mainly be considered for local control (Furuse *et al*, 1999). It has been observed that at the time of surgery, clinical N2 NSCLCs and SSTs are often even more advanced in stage than was expected preoperatively, and the complete resection rates are not sufficiently satisfactory. With the addition of radiotherapy, a greater tumour response can be expected, with the hope of better local control. Hypothetical dissemination of tumour cells during surgery may also be prevented.

## TOXICITY OF THE CHEMORADIOTHERAPY

Concurrent chemoradiotherapy is often employed as induction therapy in the trimodality approach. As compared with chemotherapy alone or sequential chemoradiotherapy, concurrent chemoradiotherapy has been associated with a higher incidence of haematologic and oesophageal toxicities (Furuse *et al*, 1999). However, the toxicity has generally been reported to be manageable, with few deaths related to treatment toxicity.

In some trials, patients undergoing definitive surgery have received a couple of courses of consolidation chemotherapy after operation. However, the compliance rate for this strategy was reported to be poor (Pisters *et al*, 2000; Rusch *et al*, 2001). In addition, some deaths related to drug toxicity have also been reported during the consolidation phase (Albain *et al*, 2005), prompting further questioning of its benefits. The role of consolidation therapy after surgery, therefore, still remains to be established.

## SURGICAL PROBLEMS IN THE TRIMODALITY APPROACH AND THEIR MANAGEMENT

Surgical resection adopted in the trimodal treatment approach is limited exclusively to a major lung resection such as lobectomy or pneumonectomy. As trimodality treatment is usually indicated for stage IIIA or IIIB disease, hilar and mediastinal lymph node dissection is also mandatory. Although preoperative chemoradiotherapy could yield better locoregional control, it would also result in a ‘frozen’ hilum and/or mediastinum. Sometimes very effective induction treatment may make it that much more difficult for the thoracic surgeon to dissect the hilum and mediastinum. Prior evaluation of the mediastinal lymph nodes by mediastinoscopy is often necessary, and this procedure also leads to a local fibrosis, which makes surgical dissection difficult. The surgical mortality associated with pneumonectomy and lobectomy without prior induction treatment has been reported to vary from 3.1 to 17.0, and 0.3 to 10.1%, respectively (Watanabe *et al*, 2004). On the other hand, when they are performed after induction chemoradiotherapy, the surgical mortality is markedly increased for both procedures (Table 1).

The ability to safely perform major lung resection following induction chemoradiotherapy would depend on several factors. Among the most important is to secure the major vessels intraoperatively. Preoperative induction treatment would melt hilar and/or mediastinal lymph nodes. As a result, these lymph nodes often become adherent to major vessels, making dissection difficult. Dissection of such lymph nodes adhering to vessels without the utmost care could lead to massive intraoperative bleeding. In such a situation, the major vessels, such as the pulmonary artery and vein, should be clamped proximally and distally to prepare for an unforeseen massive bleeding. Thoracic surgeons must not hasten to dissect such nodes. Martin *et al* (2001) reported three significant predictors of the postoperative complications: intraoperative blood loss, forced expiratory volume in the first second (percent predicted), and right pneumonectomy. Thus, thoracic surgeon must attempt their utmost to control and minimise intraoperative bleeding.

Second, the risk of bronchopleural fistula, one of the fatal complications, should be borne in mind. To prepare for minimising the risk of development of bronchopleural fistula, Stamatis *et al* (2002) recommended a reinforcement of the bronchial stump (Eberhardt *et al*, 1998). They have used thymus/mediastinal fat (89%) to cover the bronchial stump, and reported favourable results. Other materials for covering the stump include an intercostal muscle flap or diaphragmatic flap. They reported that none of their patients developed bronchopleural fistula ever since they adopted routine coverage of the bronchial stump with a vital flap. Bronchopleural fistula has been reported to develop in 0–15% of surgical patients receiving trimodality treatment. This fatal complication occurs more frequently in cases undergoing pneumonectomy, especially right pneumonectomy. Thus, coverage of the bronchial stump is strongly recommended in cases undergoing right pneumonectomy. Omentopexy may sometimes be indicated.

Finally, avoidance of postoperative pulmonary oedema is important. We usually restrict fluid administration and institute aggressive diuretic therapy postoperatively for at least first 3 days. Some recommend the use of a steroid postoperatively to avoid pulmonary oedema (Eberhardt *et al*, 1998; Stamatis *et al*, 2002).

Right pneumonectomy has been reported from various studies to be associated with very high morbidity/mortality (Table 1). Therefore, when preoperative evaluation suggests the necessity for right pneumonectomy to accomplish R0 surgery, for example, for a tumour located in the lower lobe with bulky hilar nodes, the indications for surgery must be evaluated very cautiously. Careful assessment of pulmonary function, diffusion capacity, and a quantitative ventilation scan would be necessary. Surgery should be offered only to highly selected cases, and switching to another treatment option, such as definitive chemoradiotherapy, must be seriously considered and discussed with the patient.

Bronchovascular sleeve resection has been established as one of the standard surgical procedures to spare the lung parenchyma (Deslauriers *et al*, 2004). Although highly attractive in the trimodality approach for lung cancer treatment, the feasibility of this procedure after induction chemoradiotherapy, as well as its efficacy, has yet to be confirmed.

Finally, it should be noted that surgical safety tends to be better in single-institution reports (Table 1). Therefore, methods for surgical quality control should be established for multi-institutional settings.

## SPECIFIC DISEASES

### Stage III N2 disease

#### Standard treatment for N2 NSCLC

Until the 1970s, the standard treatment for N2 NSCLC had been surgery for ‘resectable’, and definitive radiotherapy for ‘unresectable’ disease, although the treatment results remained very poor, with few long-term survivors. Neither preoperative nor postoperative radiotherapy improved the outcome.

A series of small phase III trials showed that preoperative platinum-based chemotherapy improved the survival in patients with ‘resectable’ N2 disease (Rosell *et al*, 1994; Roth *et al*, 1994). Although the results could not be reproduced in a larger randomised trial (Depierre *et al*, 2002) in the same subset of N2 patients, the role of systemic chemotherapy remains unequivocal, given the very high systemic relapse rate with local therapy alone. More recent trials have shown that postoperative adjuvant chemotherapy may improve the outcome in pathologically staged N2 patients (The International Adjuvant Lung Cancer Trial Collaborative Group, 2004; Douillard *et al*, 2006).

Platinum-based chemotherapy was also shown to improve the survival of NSCLC patients with ‘unresectable’ N2 disease, when it was added to definitive thoracic radiotherapy (Le Chevalier *et al*, 1991; Schaake-Koning *et al*, 1992; Sause *et al*, 1995; Dillman *et al*, 1996). Concurrent chemoradiotherapy was demonstrated to be superior to sequential chemoradiotherapy (Furuse *et al*, 1999). However, the optimal treatment strategy, including the roles of induction/consolidation therapy as well as the most suitable chemotherapy regimen remains undetermined.

With reports of the favourable outcome of concurrent chemoradiotherapy in stage III NSCLC cases with N2 disease, for which, therefore, this strategy now represents the standard treatment, the definition of ‘resectable’ N2 disease has become even more nebulous. In fact, surgery may not be indicated at all for clinically suspected and pathologically confirmed (via mediastinoscopy) N2 disease, even when the disease is technically ‘resectable’ (Albain *et al*, 2005).

#### Trimodality treatment for N2 NSCLC

Most of the reports of the usefulness of the trimodality approach for N2 NSCLC are based on the results of phase II or retrospective trials of preoperative chemoradiotherapy (Faber *et al*, 1989; Rusch *et al*, 1993; Deutsch *et al*, 1994; Albain *et al*, 1995; Choi *et al*, 1997; Eberhardt *et al*, 1998; Katakami *et al*, 1998; Thomas *et al*, 1999; Doddoli *et al*, 2001; Martin *et al*, 2001; Stamatis *et al*, 2002; Sonett *et al*, 2004; Cerfolio *et al*, 2005; Daly *et al*, 2006). There are some highly promising and encouraging reports of long-term survival rates of around 30%, however, the toxicity is also substantial, usually exceeding that observed with other multimodality therapy.

In view of the promising but controversial data, a few phase III trials were conducted; the ‘control’ arms of the studies were variable, reflecting the heterogeneity of N2 NSCLC, and consequently, so were the ‘concepts’ of trimodality.

In some studies, the trimodality approach appeared to derive from efforts to intensify preoperative induction chemotherapy. Before 2001, two small-scale randomised trials reported the results of trimodality *vs* induction chemotherapy for N2 NSCLC. The results of neither trial, the one showing a positive (Fleck *et al*, 1993) and the other showing a negative (Sauvaget *et al*, 2000) effect, have been published as full articles. More recently, another larger trial from Germany compared preoperative chemoradiotherapy with preoperative chemotherapy and postoperative radiotherapy, and reported no significant differences in the results (Semik *et al*, 2004). However, the actual issue should be viewed as an evaluation of the timing or sequence of the therapy, rather than as an investigation of the usefulness of the trimodality approach itself. The ‘induction chemotherapy with or without radiotherapy’ concept has now been introduced in recently initiated trials in North America and Europe.

The trimodality approach could be viewed as chemoradiotherapy followed by salvage surgery of the residual tumour. A large-scale US trial, INT0139, compared surgical resection and boost radiotherapy, in patients with N2 NSCLC receiving induction chemoradiotherapy (Albain *et al*, 2005). Although the progression-free survival (PFS) rate favoured the surgery (trimodality) arm, the overall survival rate was not statistically significantly different between the two arms, with only a marginally larger number of long-term survivors in the surgery arm.

#### Relapse pattern and its implications

Local relapse as well as emergence of distant metastases remains a big problem in N2 NSCLC patients treated by chemoradiotherapy. Concurrent chemoradiotherapy was reported to be better for local control as compared to sequential therapy (Furuse *et al*, 1999); however, the locoregional relapse rate remained high. One of the aims of the trimodality approach is to obtain better local control with surgical resection. In fact, the local relapse rate appeared to have decreased, especially in R0-surgery cases. In an intention-to-treat analysis, ‘local relapse only’ accounted for 10% of the cases showing disease progression in the trimodality arm of INT0139 (Albain *et al*, 2005), whereas it accounted for 22% of the cases showing disease progression in the chemoradiotherapy arm (*P*=0.002). However, these figures might have been biased by competitive risks; as the trimodality approach was associated with a larger number of treatment-related deaths, and as it is plausible that cases at a higher risk of postoperative morbidity/mortality were more likely suffer from uncontrollable local tumours, the local control rate with the trimodality approach could have been exaggerated by the exclusion of these high-risk patients. In addition, clinical diagnosis of failure of local control with chemoradiotherapy is often difficult because of the confounding effect of radiation fibrosis, the radiological appearance of which sometimes mimics recurrent tumours. It may not be worthwhile placing much emphasis on the difference in relapse patterns among different modalities.

One of the major problems with the use of combined modality treatment for locally advanced NSCLC is the high risk of brain metastasis (Stuschke *et al*, 1999; Thomas *et al*, 1999). A substantial proportion of patients undergoing successful R0 resection suffer from brain-only relapse. A non-randomised observational study suggested that prophylactic cranial irradiation (PCI) might reduce the risk of brain metastasis in these patients (Stuschke *et al*, 1999). Whether or not PCI would benefit optimally treated patients with locally advanced NCLC is now under investigation in randomised trials.

#### Prognostic and predictive factors

Not surprisingly, complete resection (R0 operation), which should be the aim of induction chemoradiotherapy, is associated with a better prognosis. Besides, eradication of tumour cells in the mediastinal nodes has also been reported to be a favourable prognostic factor (Choi *et al*, 1997; Albain *et al*, 2005). However, the problem is that the clinical response to induction therapy as judged by CT imaging, seems to be a poor predictor of the pathologic ‘downstaging’. There are some reports that suggest that repeated mediastinoscopy should be conducted to evaluate the status of the mediastinal nodes after induction therapy and predict the postoperative outcomes (Mateu-Navarro *et al*, 2000); however, this is not widely employed because of the technical difficulties involved. FDG-PET is currently being investigated as a promising tool for reliable assessment of the response to treatment (Pottgen *et al*, 2006).

Pathological complete response (pCR), defined as absence of viable cells in the resected specimen, has been reported to be a good prognostic factor by some, but not others. Although pCR is an established prognostic factor in patients receiving induction chemotherapy, inclusion of radiotherapy is likely to modify the information based on the locoregional response status on the effect of systemic chemotherapy against micrometastases.

Evidently, these prognostic factors, which are associated with a good response to induction therapy, would be associated with a better outcome even without subsequent surgery. Therefore, these are not necessarily predictive factors of the response to trimodality treatment, that is, it is still unclear whether surgery might be more beneficial in these patients. The only known ‘predictive’ factor of the outcome of surgical resection, which is actually a negative factor, is the necessity for pneumonectomy (Albain *et al*, 2005). An exploratory subset analysis in INT0139 revealed that patients undergoing pneumonectomy did poorly as compared to matched controls. This is clearly due to excessive early toxic deaths after pneumonectomy, in particular, right pneumonectomy or complex left pneumonectomy (see surgical problems section above). This surgical morbidity and mortality might well offset the advantage of PFS in the trimodality arm.

### Superior sulcus tumours

Superior sulcus tumours have long been treated by preoperative radiation and resection, but both the curative resection (R0) rate (50%) and the long-term survival rate (30%) have remained poor and unchanged over the last 40 years (Rusch *et al*, 2001). Two large-scale phase II trials from the US and Japan (Rusch *et al*, 2001; Kunitoh *et al*, 2003) suggested that preoperative concurrent chemoradiotherapy could improve the outcome in these patients. In both series, the R0 rate was approximately 70%, with a 5-year survival rate of 40–50%. The treatment-related death rate was acceptably low at 4%.

Because of the infrequent occurrence of this tumour subtype, a randomised trial would be extremely difficult. Therefore, given the reproducibility of the favourable results as described above, the trimodality approach may be accepted as the standard treatment strategy for SSTs.

The relatively low morbidity/mortality of trimodality treatment in patients with SSTs would be ascribed to the small size of the irradiation field, which is a known predictive factor for radiation toxicity. Theoretically, it would allow escalation of the radiation dose, however, intensification of chemotherapy and not radiotherapy should be the next logical step for further improvement of the results, because the relapse pattern in these cases is predominantly represented by distant metastases (Rusch *et al*, 2001; Kunitoh *et al*, 2003). Brain metastasis is also a challenging problem in cases with SSTs, and PCI may have a role in the management of these patients.

### T4 tumours (other than SSTs)

Induction chemoradiotherapy might be beneficial for patients with other T4 diseases, which invade the major vessels or organs. Clinical response to the induction therapy may allow the surgeons to resect the initially unresectable tumour. However, surgical resection of T4 lung cancer, involving the carina, atrium, and/or vena cava, is technically challenging, and surgical morbidity is still high even for surgery alone (Tsuchiya *et al*, 1994). Grunenwald *et al* (2001) reported a surgical mortality after chemoradiotherpy of 7% and 5-year survival rate of 19%. As mediastinal cleaning by induction therapy was reported to be prognostic, patients with T4N0-1 disease might be good candidates for an attempt of this approach. However, this remains to be confirmed in a prospective study.

## SUMMARY AND CONCLUSIONS

### Optimal management of patients undergoing trimodality treatment

**Figure 1:**
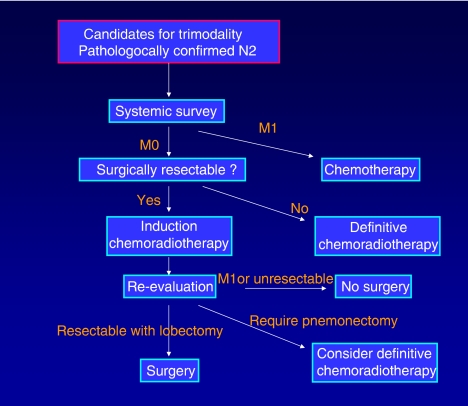
Management of N2 disease patients potentially indicated for trimodality.

**Figure 2:**
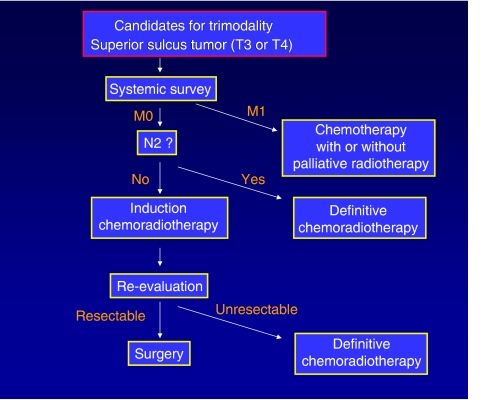
Management of SST patients potentially indicated for trimodality.

The trimodality approach, although it is sometimes highly effective, poses substantial risks to the patients. The risk/benefit ratio must be carefully evaluated on an individual basis (Figures 1 and 2).

As the candidates for this treatment approach have locally advanced disease, potential distant metastasis should be excluded by complete staging, preferably by methods including brain MRI and whole-body PET scans, before the initiation of therapy. After the induction therapy, in addition to re-staging of the local disease by CT imaging, brain MRI should be repeated, considering the high risk of brain metastasis.

Although downstaging of the tumour after induction chemoradiotherapy sometimes does occur, physicians should not enrol patients with technically unresectable disease at presentation, with the hope of conversion of unresectable to resectable disease after induction therapy. The exception, of course, is SST, in which apparently unresectable T4 disease at presentation does not represent a contraindication to trimodality treatment aimed at complete resection (Rusch *et al*, 2001; Kunitoh *et al*, 2003). There are no data on the results of trimodality treatment in cases of SSTs with N2 disease, therefore, this approach cannot yet be recommended for these patients at present.

Right pneumonectomy after induction chemoradiotherapy has been reported to be associated with unacceptably high surgical morbidity/mortality (Albain *et al*, 2005), and patients in whom a right pneumonectomy for R0 resection would be indicated should be very cautiously evaluated to determine whether or not they might be suitable candidates for the trimodality approach. Physicians should also be discouraged from considering the trimodality approach for such a patient in the hope of downstaging of the tumour with induction therapy.

### Conclusions

For locally advanced NSCLC patients with clinical N2 disease, the trimodality approach, although promising, should still be considered as investigational therapy. The suitability of a given patient for this therapy must be meticulously determined by surgeons, radiation oncologists, medical oncologists and chest physicians. For SSTs, the trimodality approach can now be considered as standard therapy, but it should be managed by an experienced multimodality team as the risk remains substantial, with a treatment-related death rate of 4%. Enrolment of patients into clinical trials is strongly encouraged as there still remain numerous unanswered questions.

## Figures and Tables

**Table 1:** Surgical morbidity and overall efficacy in trimodal treatment

					**Induction therapy**						**Postoperative morbidity**		**Bronchopleural fistula**		**Postoperative surgical mortality**			
**Author**	**Study participants**	**Study design**	**Inclusion criteria**	**No. of cases**	**CT**	**RT(Gy)**	**Mode**	**Resection rate**	**No. of surgical patients**	**No. of pneumo**	**Overall**	**Pneumo**	**Overall**	**Pneumo**	**Overall**	**Pneumo**	**Right pneumo**	**Survival rate**
Deutsch *et al* (1994)	Multi-institution	Phase II	Stage IIIA	28	Carbo/VP16	60	C	43%	16	6	6 (37.5%)	3 (50%)	1 (6.3%)	—	3 (18.7%)	2 (33.3%)	NR	NR
Faber *et al* (1989)	Single institution	Phase II	Stage III	85	Cis/5-FU/VP16	40	C	73%	62	24	14 (21.9%)	6 (24%)	4 (6.5%)	3 (12.5%)	3 (5%)	3 (8.9%)	NR	40% (3 years)
Albain *et al* (1995, 2005) Rusch *et al* (2001)	Multi-institution	Phase II	T4, N2, N3	126	Cis/VP16	45	C	86%	107	38	NR	NR	NR	NR	8 (7.5%)	6 (15.8%)	NR	24-27% (3 years)
Katakami *et al* (1998)	Multi-institution	Phase II	Stage III	42	Cis/VP16	50	C	45%	19	10	NR	NR	1 (5.3%)	1 (10%)	2 (11%)	2 (20%)	1 (10%)	11.4% (5 years)
Thomas *et al* (1999)	Single institution	Phase II	T4, N2, N3	54	Ifo/Carbo/VP16	45	H	63%	40	15	NR	NR	4 (10%)	NR	4 (10%)	3 (20%)	NR	30% (3 years)
Choi *et al* (1997)	Single institution	Phase II	N2 stage IIIA	42	Cis/vinb/5-FU	42	H	93%	39	7	10 (23.8%)	NR	0	0	2 (5%)	0	0	37% (5 years)
Sonett *et al* (2004)	Single institution	Retrospective	N/A	40	Platinum based	62	C	N/A	40	11	7 (17.5%)	NR	1 (2.5%)	1 (9.1%)	0 (0%)	0 (0%)	0 (0%)	46% (5 years)
Martin *et al* (2001)	Single institution	Retrospective	N/A	470	Platinum based	10–72	C	N/A	470	97	179 (38.1%)	45 (46.4%)	8 (1.7%)	NR	18 (3.8%)	11 (11.3%)	11 (23.9%)	NR
Eberhardt *et al* (1998) Stamatis *et al* (2002)	Single institution	(Phase II)^a^ Retrospective	N2, N3	350	Cis/Tax Cis/VP16	45	H	(53%)^a^	350	125	154 (44%)	NR	14 (4.0%)	10 (8.0%)	17 (4.9%)	9 (7.2%)	NR	(31% (4 years))^a^
Doddoli *et al* (2001)	Single institution	Retrospective	N/A	69	Platinum based	20–60	C	N/A	69	33	12 (17%)	7 (21%)	5 (15%)	5 (15%)	6 (9%)	3 (9.0%)	3 (13.6%)	NR
Daly *et al* (2006)	Single institution	Retrospective	N/A	30	Platinum based	60	C	N/A	30	30	5 (16.6%)	5 (16.6%)	1 (3.3%)	1 (3.3%)	4 (13.3%)	4 (13.3%)	1 (5.6%)	38% (5 year)
Cerfolio *et al* (2005)	Single institution	Retrospective	N/A	104	Carbo based	45–66.7	C	N/A	104	12	21 (20.1%)	7 (58.3%)	1 (1.0%)	NR	3 (2.9%)	2 (16.7%)	1 (14%)	30-38% (4 year)
Albain *et al* (1995, 2005)	Multi-institution	Phase III	N2 stage IIIA	202	Cis/VP16	45	C	71%	164	54	NR	NR	NR	NR	16 (7.9%)	14 (25.9%)	11 (37.9%)	27% (5 year)
Semik *et al* (2004)	Single institution	Phase III	Stage III	179	Cis/VP16 Carbo/Vind	45	H	58%	130	43	NR	NR	5 (3.8%)	NR	8 (6.2%)	NR	NR	NR

Abbreviations: CT, chemotherapy; RT, radiotherapy; pneumo, pneumonectomy; Carbo, carboplatin; Cis, cisplatin; VP16, etoposide; vinb, vinblastine; 5-FU, 5-fluorouracil; Ifo, ifosfamide; Tax, paclitaxel; C, conventional; H, hyperfractionated; N/A, not applicable; NR, not reported.

a In prospective phase II trial (Eberhardt *et al*, 1998).
